# A descriptive analysis of spontaneous reports of antipsychotic‐induced tardive dyskinesia and other extrapyramidal symptoms in the Japanese Adverse Drug Event Report database

**DOI:** 10.1002/npr2.12385

**Published:** 2023-10-26

**Authors:** Yosuke Saga, Chih‐Lin Chiang, Akihide Wakamatsu

**Affiliations:** ^1^ Medical Affairs Division Janssen Pharmaceutical K.K Tokyo Japan

**Keywords:** antipsychotic, extrapyramidal symptoms, JADER, pharmacovigilance, tardive dyskinesia

## Abstract

**Aim**The aim of this study is to summarize the spontaneous reports of tardive dyskinesia (TD) and extrapyramidal symptoms (EPSs) that occurred in Japan over the past decade.

**Methods**The study analyzed TD and EPS cases reported in the Japanese Adverse Drug Event Report database between April 2011 and March 2021. The cases were stratified by the diagnoses of schizophrenia, bipolar disorders, and depressive disorders.

**Results**In total, 800 patients including a total of 171 TD cases and 682 EPS cases were reported in the JADER database across psychiatric diagnosis. The cases were caused by first‐generation antipsychotics (FGA, TD: *n* = 105, EPS: *n* = 245) and second‐generation antipsychotics (SGA, TD: *n* = 144, EPS: *n* = 598). The SGA were categorized based on Neuroscience‐based Nomenclature (NbN) regarding pharmacological domain and mode of action, which were reported evenly as the offending agents. Among reported treatment and outcome in TD cases (*n* = 67, 37.6%) and EPS cases (*n* = 405, 59.3%), the relatively limited number of TD cases were reported as recovered/improved was also limited (*n* = 32, 47.8%) compared to those of EPS cases (*n* = 266, 65.7%). Some cases still had residual symptoms or did not recover fully (TD: *n* = 21, 31.3%, EPS: *n* = 77, 19.0%).

**Conclusion:**

Tardive dyskinesia and EPS have been widely reported in Japan over the past decade across psychiatric diagnoses and antipsychotic classes.

**Limitations:**

It is important to acknowledge the presence of reporting bias and the lack of comparators to accurately assess risks. Owing to the nature of spontaneous reporting, the estimation of prevalence is not feasible.

## Abstract 2

This study is a summary of the spontaneous reports of tardive dyskinesia (TD) and extrapyramidal symptoms (EPSs) in Japanese clinical practice during the last decade. This study demonstrates that TD and EPS have been widely reported in Japan over the past decade across psychiatric diagnoses including schzophrenia, bipolar disorder, and mood disorder, regardless of antipsychotic classes.

## Graphical abstract

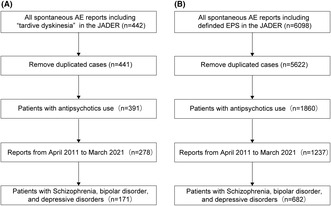

## 1 INTRODUCTION

Tardive dyskinesia (TD), a severe symptoms that develops after long‐term exposure to dopamine‐blocking antipsychotics,^1^ can negatively affect the treatment of the primary disorder and reduce a patient's activity of daily life (ADL) and Quality of Life (QoL).^2^, ^3^

Several risk factors for TD have been identified, including older age, female gender, longer use of antipsychotics, higher dosages of antipsychotics, a history of extrapyramidal symptoms (EPSs), and the use of first‐generation antipsychotics (FGAs).^4^, ^5^, ^6^, ^7^, ^8^, ^9^, ^10^ Although the risk of TD with second‐generation antipsychotics (SGAs) is lower compared with FGAs,^11^ it still exsists.^12^ In addition, polypharmacy, or the use of more than two antipsychotics over a prolonged period, increases the risk of TD and EPS due to higher dosages and use of anticholinergic drugs.^13^ Furthermore, patients with mood disorders who receive antipsychotics are at increased risk for TD^7^, ^14^ and up to half of patients with bipolar disorder receive antipsychotics.^15^ However, there is limited evidence about TD in Japan.^16^

This study aimed to answer the following questions addressed: (1) how many patients with TD and EPS have been reported in Japan over the past decade, and in which psychiatric diagnoses? (2) which antipsychotic classes of has been reported as offending agents for TD? (3) Does TD improve adequately with current treatment? To address these questions, we analyzed the Japanese Adverse Drug Event Report (JADER) database, published by the Pharmaceuticals and Medical Devices Agency (PMDA) in Japan. JADER collects spontaneous AE reports in Japan, making it the most suitable database to identify TD cases and analyze them. Our analysis included both TD and EPS cases, as EPS is a risk factor for TD.

## 2 MATERIALS AND METHODS

The JADER database is a valuable resource for post‐marketing surveillance and spontaneous reports of adverse events (AEs) related to medication use. The database is compiled by the PMDA, a Japanese regulatory authority established by the Ministry of Health, Labour and Welfare in 2004. Healthcare professionals and patients can report AEs through the spontaneous reporting system. The data in JADER is fully anonymized by the PMDA and can be accessed through their website (www.pmda.go.jp). The use of JADER has been described in detail in previous studies.^17^

The JADER database adheres to the guidelines set by the International Conference for Harmonization (ICH) for the registration of pharmaceuticals for human use and provides standardized information on AEs. The database includes details for each AE case, such as the patient's age, gender, the name of the medicinal product/substance causing the AE, the nature of the AE, and the outcome of the case. The information is collected through spontaneous reporting from physicians, pharmacists, other healthcare professionals, and customers such as caregivers, patients and their family, and is maintained by the PMDA. The data is fully anonymized by PMDA to maintain privacy. Note that because JADER is a spontaneous reporting system, there is possibility that reported cases could be underreported compared to clinical practice.

### 2.1 Analysis

**FIGURE 1:**
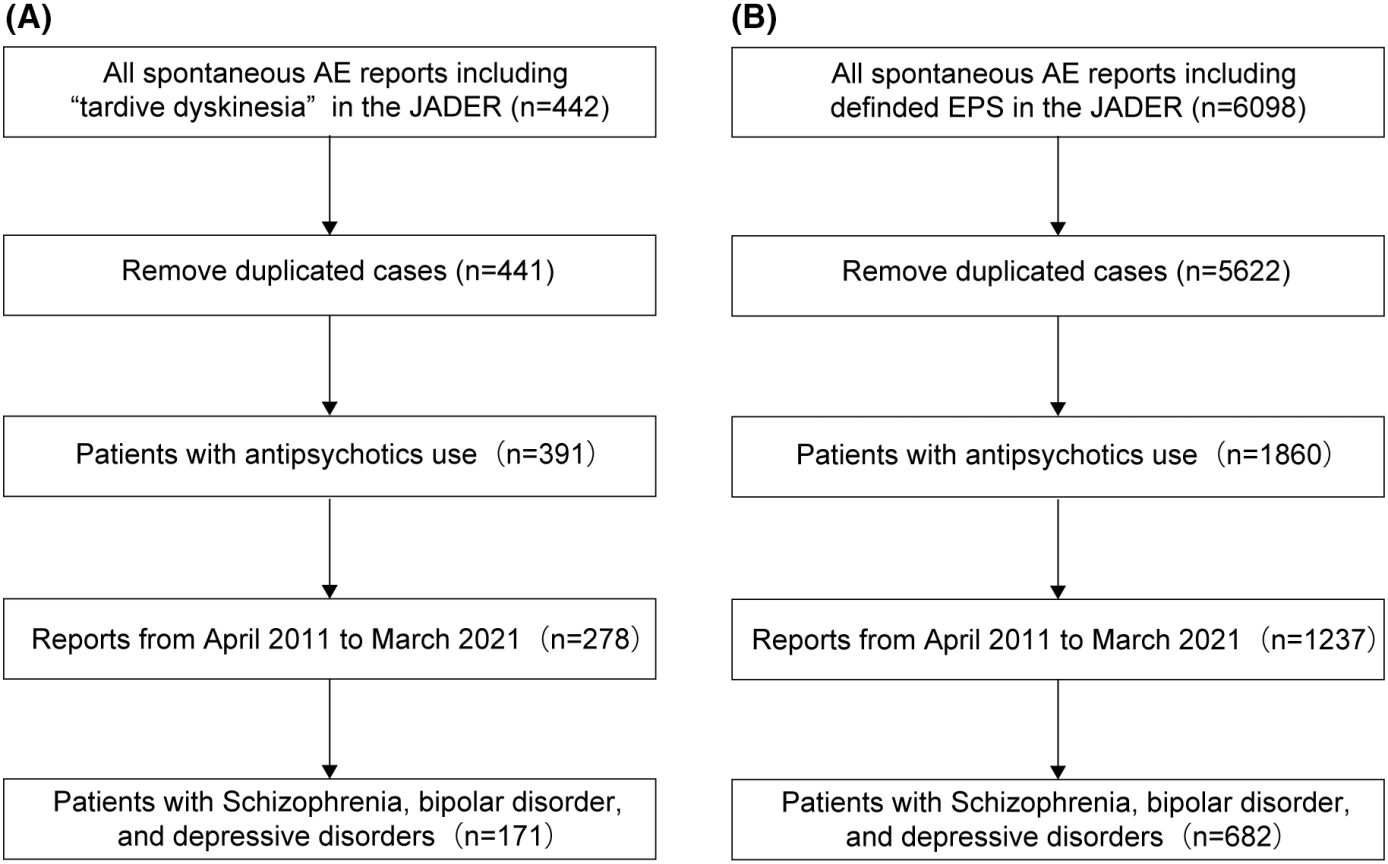
Patient record selection flow. Patient selection flow for tardive dyskinesia (TD) cases (A) and extrapyramidal symptoms (EPSs) cases (B). Duplicated cases were identified by patient ID in the data sheet.

To understand the occurrence of TD and EPS associated with prescribed antipsychotics drug category, we analyzed the JADER. The data were extracted and analyzed from April 2011 to March 2021 which would reflect recent prescription trend (Figure 1). The database includes information on patients' demographics, prescribed antipsychotic class, and reported side effects. The number of patients and information on monotherapy and polytherapy were counted based on the purpose of use information, and if the purpose of use information was missing, the data were referenced by the diagnosis information.

We focused on records related to schizophrenia, bipolar disorder, and depressive disorder, since antipsychotics are mainly prescribed to these diseases. In order to identify diagnosed diseases, we extracted reported data associated with specific diagnosis or marked as below: schizophrenia includes schizophrenia, schizoaffective disorder, schizophreniform disorder, and schizotypal (personality) disorder, bipolar disorder includes bipolar disorder, bipolar I disorder, and bipolar II disorder, depressive disorder includes depressive disorder with suicidal ideation, depressive disorder with peripartum onset, depressive disorder with insomnia disorder, depressed mood, depressed symptom, persistent depressive disorder, antidepressant therapy, and postoperative depression.

To analyze the use of antipsychotics, we classified the prescribed drugs into FGA and SGA available in Japan (Table S1). The SGA groups were further categorized based on Neuroscience‐based Nomenclature (NbN, https://www.cinp.org/nomenclature) regarding pharmacological domain and mode of action: Group1: antagonist of dopamine and serotonin (i.e. Blonanserin, Lurasidone, Olanzapine, Perospirone, and Zotepine), Group 2: antagonist of dopamine, serotonin, and norepinephrine (i.e. Asenapine, Clozapine, Paliperidone, and Risperidone), Group 3: multimodal agent of dopamine and serotonin and norepinephrine (i.e. Quetiapine), Group 4: partial agonist of dopamine and serotonin (i.e. Aripiprazole and Brexpiprazole, Table S1). Note that some patients were prescribed multiple antipsychotics (i.e. polypharmacy), resulting in the number of prescribed antipsychotics exceeding the number of patients. We extracted most common EPS manifestations^18^ as follows: akathisia, dystonia, dyskinesia, akinesia, bradykinesia, dysarthria, cogwheel rigidity, parkinsonism, tremor, parkinsonian gait, and hypersalivation.

We counted the number of TD and EPS cases, and based on gender, age group, and antipsychotic class in each psychiatric disease. Furthermore, we investigated the association between management of antipsychotics dosing (i.e. discontinue, no change, add medication, dosage reduction, unknown) and it's outcome (recovered, improved, residual symptom, not recovered, death, unknown). These reports were decided by physicians without specific definition. Then, we counted number of patients who experienced TD and each EPS based on antipsychotic category and diagnosis. In addition, we counted the number of patients who had comorbid TD and EPS (i.e. comorbidity).

## 3 RESULTS

### 3.1 Patients' demographic in reported TD and EPS

The majority of reporters were health care professionals, including physicians, pharmacists, and medical staff, accounting for 84% of all reporters. The remaining reporters were from customers (14.0% of TD cases and 10.8% of EPS cases), with 2.8% of TD cases and 1.5% of EPS cases being attributed to unknown sources. A total of 800 patients were reported with 171 TD cases and 682 EPS patients (Figure 1, Table 1). Of the TD cases, 123 cases in patient with schizophrenia, 20 in those with bipolar disorder, and 35 in patient with depressive disorder. The numbers of reported EPS cases were 427 in patients with schizophrenia, 98 in those with bipolar, and 211 in those with depressive disorder cases during the study period (Table 1).

**TABLE 1:** Patient demographics and antipsychotic medication treatment in the adverse event report.

	TD				EPS			
	Overall	Schizophrenia^a^	Bipolar^a^	Depression^a^	Overall	Schizophrenia^a^	Bipolar^a^	Depression^a^
*N*	178	123	20	35	736	427	98	211
Sex, *n* (%)								
Male	91 (51.1)	65 (52.9)	12 (60.0)	14 (40.0)	312 (42.4)	198 (46.4)	40 (40.8)	74 (35.0)
Female	87 (48.9)	58 (47.2)	8 (40.0)	21 (60.0)	418 (56.8)	226 (52.9)	57 (58.1)	135 (64.0)
Unknown	0 (0.0)	0 (0.0)	0 (0.0)	0 (0.0)	6 (0.8)	3 (0.7)	1 (1.0)	2 (0.9)
Age group, *n* (%)								
<20 years	3 (1.7)	3 (2.4)	0 (0)	0 (0)	22 (3.2)	19 (4.5)	0 (0)	3 (1.8)
20–39 years	48 (27.0)	34 (27.6)	10 (50.0)	4 (11.4)	169 (24.9)	123 (29.1)	21 (24.4)	25 (14.6)
40–59 years	67 (37.6)	45 (36.6)	6 (30.0)	16 (45.7)	261 (38.4)	168 (39.7)	29 (33.7)	64 (37.4)
60–79 years	53 (29.8)	36 (29.3)	4 (20.0)	13 (37.1)	208 (30.6)	87 (20.6)	41 (47.7)	80 (46.8)
≥80 years	3 (1.7)	2 (1.6)	0 (0)	1 (2.9)	39 (5.7)	8 (1.9)	3 (3.5)	28 (16.4)
Unknown	4 (2.2)	3 (2.4)	0 (0)	0 (0)	37 (5.4)	22 (5.2)	4 (4.7)	11 (6.4)
AP treatment at the time of report (all formulations), *n* (%)								
*N*	175	123	20	32	702	425	91	186
AP monotherapy	64 (36.6)	38 (30.9)	6 (30.0)	20 (62.5)	364 (51.9)	182 (42.8)	47 (51.6)	135 (72.6)
AP polytherapy	111 (63.4)	85 (69.1)	14 (70.0)	12 (37.5)	338 (48.1)	243 (57.2)	44 (48.4)	51 (27.4)
Any FGA	105 (60.0)	66 (53.7)	13 (65.0)	26 (81.3)	245 (34.9)	119 (28.0)	19 (20.9)	107 (57.5)
Any SGA	144 (82.3)	111 (90.2)	18 (90.0)	15 (46.9)	598 (85.2)	410 (96.5)	87 (95.6)	101 (54.3)
Group 1	83 (57.6)	64 (57.7)	13 (72.2)	6 (40.0)	259 (43.3)	175 (42.7)	52 (59.8)	32 (31.7)
Group 2	72 (50.0)	59 (53.2)	11 (61.1)	2 (13.3)	287 (48.0)	248 (60.5)	17 (19.5)	22 (21.8)
Group 3	46 (31.9)	35 (31.5)	4 (22.2)	7 (46.7)	141 (23.6)	86 (21.0)	26 (29.9)	29 (28.7)
Group 4	66 (45.8)	58 (52.3)	3 (16.7)	5 (33.3)	237 (39.6)	160 (39.0)	34 (39.1)	43 (42.6)
AP treatment at the time of report (oral and injectable formulations only), *n* (%)								
*N*	163	118	17	28	619	403	85	146
AP monotherapy	59 (36.2)	36 (30.5)	6 (35.3)	17 (60.7)	317 (50.0)	170 (42.2)	43 (50.6)	104 (71.2)
AP polytherapy	104 (63.8)	82 (69.5)	11 (64.7)	11 (39.3)	317(50.0)	233 (57.8)	42 (49.4)	42 (28.8)
Any FGA	93 (57.1)	61(51.7)	10(58.8)	22(78.6)	198(32.0)	102(25.3)	18(21.2)	78(53.4)
Any SGA	128 (78.5)	99 (83.9)	15 (88.2)	14 (50.0)	551 (89.0)	386 (95.8)	81 (95.3)	84 (57.5)
SGA subclass	235	194	24	17	829	614	115	100
Group 1	75 (31.9)	59 (30.4)	10 (41.7)	6 (35.3)	241 (29.1)	165 (26.9)	50 (43.5)	26 (26.0)
Group 2	57 (24.3)	48 (24.7)	7 (29.2)	2 (11.8)	248 (29.9)	222 (36.2)	13 (11.3)	13 (13.0)
Group 3	44 (18.7)	34 (17.5)	4 (16.7)	6 (35.3)	127 (15.3)	80 (13.0)	22 (19.1)	25 (25.0)
Group 4	59 (25.1)	53 (27.3)	3 (12.5)	3 (17.6)	213 (25.7)	147 (23.9)	30 (26.1)	36 (36.0)

*Note*: Group 1: antagonist of dopamine and serotonin, Group 2: antagonist of dopamine, serotonin, and norepinephrine, Group 3: multimodal agent of dopamine and serotonin and norepinephrine, Group 4: partial agonist of dopamine and serotonin.

Abbreviations: AP, antipsychotic; EPSs, extrapyramidal symptoms; FGA, first‐generation antipsychotics; SGA, second‐generation antipsychotics; TD, tardive dyskinesia.

^a^ A case can be classified into multiple diagnostic categories if multiple diagnostic codes were recorded in the adverse event report.

Among TD cases, 51 (28.6%) were also reported as EPS. Dystonia was most common reported, with 34 cases reported. Other reported EPS included dyskinesia and dystonia (*n* = 8), parkinsonism (*n* = 5), tremor (*n* = 1), dyskinesia & dysarthria (*n* = 1), dystonia & dyskinesia & dysarthria (*n* = 1), and akathisia & bradykinesia & parkinsonism (*n* = 1). Regardless of the reported disorder, dystonia, parkinsonism, akathisia, and dyskinesia were commonly reported among the EPS as listed in Table S2.

### 3.2 Antipsychotics categories associated with TD and EPS

Next, we analyzed the prescription of antipsychotics as either monotherapy or polytherapy (Table 1). As for TD, 111 (63.4%) reports were polytherapy (monotherapy: *n* = 64, 36.6%). On the other hand, monotherapy and polytherapy were evenly reported for EPS (monotherapy: *n* = 364, 51.9%, polytherapy: *n* = 338, 48.1%). The number of reported prescribed antipsychotics as offending agents showed that SGA cases were larger than FGA cases (SGA: 144, 82.3% TD cases, and 598, 85.2% EPS cases, FGA: 105, 60.0% TD cases and 245, 34.9% EPS cases). This included 83 (57.6%) TD and 259 (42.7%) EPS cases associated with Group 1, 72 (50.0%) TD and 287 (48.0%) EPS cases associated with Group 2, 46 (31.9%) TD and 141 (23.6%) EPS cases associated with Group 3, and 66 (45.8%) TD and 237 (39.6%) EPS cases associated with Group 4 (Table 1).

**FIGURE 2:**
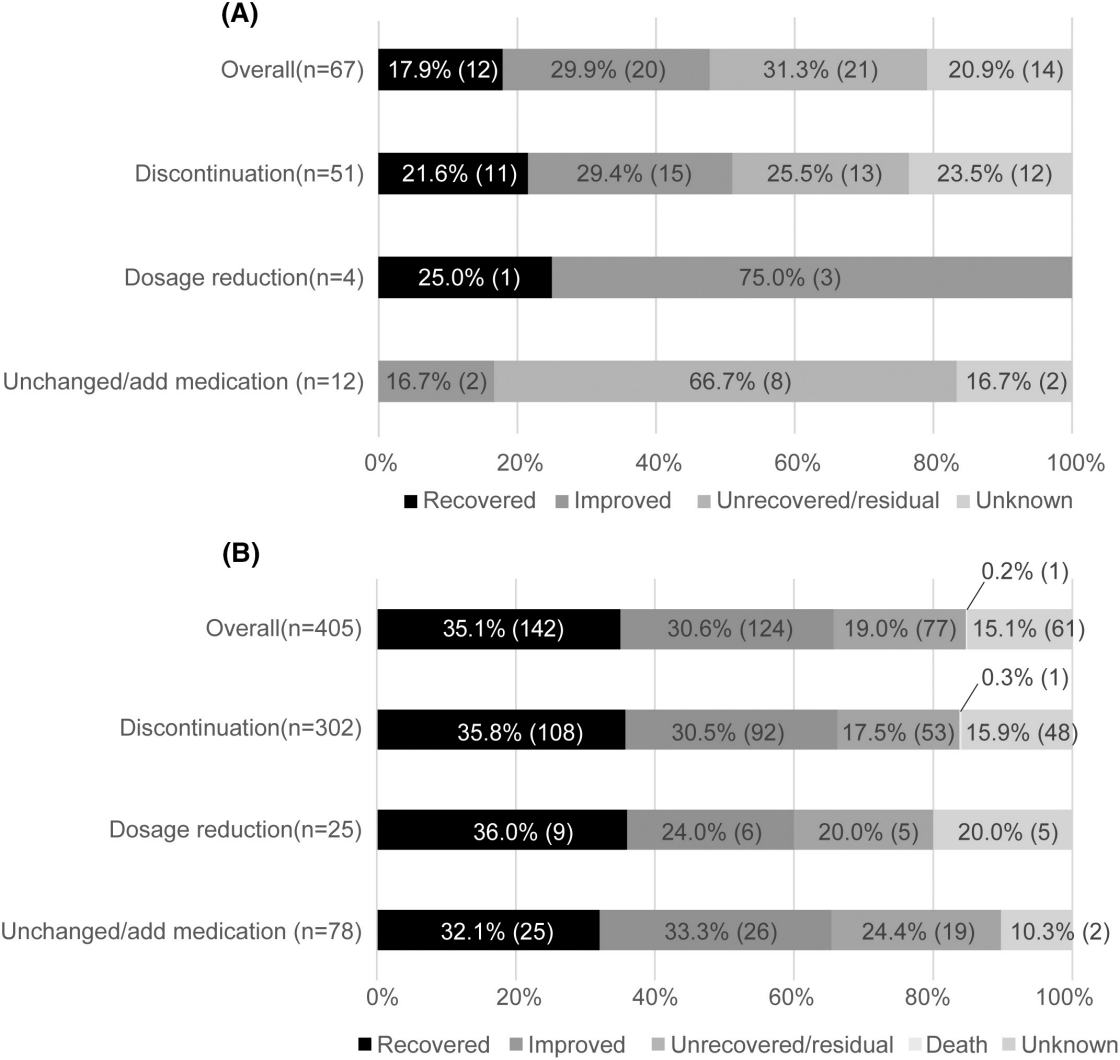
The association between treatment and its outcome in patients with tardive dyskinesia (TD) (A) and extrapyramidal symptoms (EPSs) (B). Among TD reports (*n* = 171) and EPS (*n* = 680), 67 and 405 cases were reported its treatment. Treatments were categorized as discontinuation/dosage reduction, unchanged, and adding medication. Numbers in parentheses indicate the number of reports.

Among the 171 TD cases and 682 EPS cases, 67 (39.2%) and 405 (59.3%) had recorded both treatment and outcome information (Figure 2). Out of TD cases, 12 (17.9%) recovered, 20 (29.9%) improved, and 21 (31.3%) remained residual or unrecovered, with 14 cases (20.9%) were unknown. Discontinuation of treatment was taken in 51 cases (76.1%), resulting in 11 (21.6%) recoveries and 15 (29.4%) improvements. Of the 12 cases (17.9%) where medication was unchanged or increased, 8 (66.7%) remained residual or unrecovered, while 4 (16.7%) of the cases with reduced dosage showed improvement (*n* = 1, 25.0%) or recovery (*n* = 3, 75.0%). One TD case was reported as a death (data not shown). In the case of EPS, 142 cases (35.1%) showed recovery, and 124 cases (30.6%) exhibited improvement (Figure 2B). The most administered treatments included discontinuation (*n* = 302, 74.6%), followed by unchanged/added medication (*n* = 78, 19.3%), which resulted in more than 65% of cases experiencing recovery or improvement. The relative proportions of recovered/improved showed significantly different between TD cases and EPS cases (Ficher's exact test, *p* = 0.0105, two‐sided test).

## 4 DISCUSSION

Our study has revealed that (1) TD and EPS were reported not only patients with schizophrenia but also patients with bipolar disorder and depressive disorders, (2) offending agents were both SGA and/or FGA including polypharmacy and monotherapy, and (3) some patients were non‐recovered in TD cases even after their current antipsychotic treatment was discontinued.

The number of reported TD cases, the relative number of reported cases were larger in patients treated with SGA compared to FGA, reflecting the current trend in Japan where SGA is the widely prescribed as the preferred treatment option.^19^ The antipsychotics responsible for inducing TD were largely in line with prior reports.^19^, ^20^ The relationship between formulation (oral or long‐acting injectable; LAI) and TD occurrence has been reported, with SGA–LAIs being less likely to induce TD compared to oral antipsychotics and FGA–LAIs.^21^ However, further longitudinal studies are needed to fully understand the relationship between formulation and TD.

The Japanese Society of Neuropsychopharmacology recommends reducing or discontinuing the offending agents to improve TD.^22^ However, previous studies have indicated that TD is often irreversible and that discontinuing the offending agent only leads to resolution in a limited number of patients.^1^, ^23^ There is limited evidence regarding the effectiveness of reducing the dosage as a treatment for TD.^24^ Moreover, the necessity of a minimal antipsychotic dose to control unstable symptoms and maintain a stable condition would not allow for a change or switch of antipsychotic in clinical practice.^25^ As a result, some TD cases remain unrecovered, suggesting that discontinuation or dosage reduction may not be an effective treatment for TD. It was found that approximately 65% of EPS cases recovered or improved. Conversely, roughly 48% of TD cases recovered or improved (Figure 2). This result may imply the challenge of the management of TD.

### 4.1 Limitation

Reports made to the database are not restricted to medical professionals and can come from anyone, including care givers and patient's family etc., leading to the possibility of biased information being recorded. However, in this study, over 80% of the cases were reported by physicians or medical staff and the analyzed data was comprehensive. Second, since JADER only collects incidence of AE events, with no information about the severity of symptoms, disease duration, duration of antipsychotic treatment, nor treatment history which are clinically important factors to be considered. Finally, considering the absence of a comparator, the estimation of relative risk is not achievable.

## 5 CONCLUSION

Tardive dyskinesia and EPS have been widely reported across various diseases and antipsychotic classes in Japan in the past decade. There are limited number of TD cases reported as recovered even after discontinuation of antipsychotics. These findings suggest that TD is not sufficiently treated and a significant burden to patients and call for more effective therapeutic options to address these conditions.

## AUTHOR CONTRIBUTIONS

Study design and concept: Y. S., A. W., analyzed data: Y. S., writing manuscript: Y. S., Review the draft: Y. S., A. W., and C.‐C. L.

## FUNDING INFORMATION

This study was supported by Janssen Pharmaceutical K.K.

## CONFLICT OF INTEREST STATEMENT

All the authors are employees of Janssen Pharmaceutical K.K.

## ETHICS STATEMENT

Approval of the Research Protocol by an Institutional Reviewer Board: The study protocol has been approved by internal review board in Janssen Pharmaceutical K.K.

Informed Consent: N/A.

Registry and the Registration No. of the Study/Trial: N/A.

Animal Studies: N/A.

## Supporting information


Tables S1–S2.

## Data Availability

Data derived from public domain resources JADER published by PMDA (URL: https://www.pmda.go.jp/safety/info‐services/drugs/adr‐info/suspected‐adr/0003.html).
