# Supplementary material for: A descriptive analysis of spontaneous reports of antipsychotic‐induced tardive dyskinesia and other extrapyramidal symptoms in the Japanese Adverse Drug Event Report database
Source: Neuropsychopharmacol Rep. 2023 Oct 26;44(1):221–6. doi: 10.1002/npr2.12385 (PMC10932779; doi:10.1002/npr2.12385)
Supplement: Supplementary file 1 — Tables S1–S2. [file NPR2-44-221-s001.docx]

| Compound | FGA/SGA | Neuroscience-based nomenclature (NbN) | | SGA group |
| --- | --- | --- | --- | --- |
|  |  | Pharmacology | Mode of Action |  |
| Bromperidol | FGA | - | antagonist | - |
| Chlorpromazine | FGA | dopamine, serotonin | antagonist | - |
| Clocapramine | FGA | - | antagonist | - |
| Fluphenazine | FGA | dopamine | antagonist | - |
| Haloperidol | FGA | dopamine | antagonist | - |
| Levomepromazine | FGA | - | antagonist | - |
| Mosapramine | FGA | - | antagonist | - |
| Nemonapride | FGA | - | antagonist | - |
| Perphenazine | FGA | dopamine | antagonist | - |
| Pimozide | FGA | dopamine | antagonist | - |
| Propericiazine | FGA | - | - | - |
| Sulpiride | FGA | dopamine | antagonist | - |
| Sultopiride | FGA | - | - |  |
| Timiperone | FGA | - | - | - |
| Blonanserin | SGA | dopamine, serotonin | antagonist | Group1 |
| Lurasidone | SGA | dopamine, serotonin | antagonist | Group1 |
| Olanzapine | SGA | dopamine, serotonin | antagonist | Group1 |
| Perospirone | SGA | dopamine, serotonin | antagonist | Group1 |
| Zotepine | SGA | dopamine, serotonin | antagonist | Group1 |
| Asenapine | SGA | dopamine, serotonin, norepinephrine | antagonist | Group2 |
| Clozapine | SGA | dopamine, serotonin, norepinephrine | antagonist | Group2 |
| Paliperidone | SGA | dopamine, serotonin, norepinephrine | antagonist | Group2 |
| Risperidone | SGA | dopamine, serotonin norepinephrine | antagonist | Group2 |
| Quetiapine | SGA | dopamine, serotonin, norepinephrine | multimodal | Group3 |
| Aripiprazole | SGA | dopamine, serotonin | partial agonist | Group4 |
| Brexpiprazole | SGA | dopamine, serotonin | partial agonist | Group4 |

Sup. Table.1 List of antipsychotics categories

Group1: antagonist of dopamine and serotonin,

Group2: antagonist of dopamine, serotonin, and norepinephrine,

Group3: multimodal agent of dopamine and serotonin and norepinephrine,

Group4: partial agonist of dopamine and serotonin

-: not available in NbN

|  | SCH | | | | | Bipolar | | | | | Depressive | | | | |
| --- | --- | --- | --- | --- | --- | --- | --- | --- | --- | --- | --- | --- | --- | --- | --- |
|  | Dystonia | Parkinsonism | Akathisia | Dyskinesia | Hypersalivation | Parkinsonism | Dystonia | Tremor | Akathisia | Dyskinesia | Parkinsonism | Tremor | Dyskinesia | Dystonia | Akathisia |
| N | 141 | 96 | 61 | 57 | 39 | 37 | 20 | 13 | 16 | 10 | 87 | 23 | 35 | 36 | 27 |
| Sex, n (%) | | | | | | | | | | | | | | | |
| male | 72 (51.1) | 42 (43.8) | 18 (29.5) | 28 (49.2) | 25 (64.1) | 14 (37.8) | 10 (50.0) | 5 (38.5) | 4 (25.0) | 6 (60.0) | 28 (32.2) | 6 (26.1) | 12 (34.3) | 19 (52.8) | 7 (25.9) |
| female | 69 (48.9) | 54 (56.3) | 42 (68.9) | 28 (49.1) | 13 (33.3) | 23 (62.2) | 9 (45.0) | 8 (61.5) | 12 (75.0) | 4 (40.0) | 59 (67.8) | 17 (73.9) | 23 (65.7) | 15 (41.7) | 20 (74.7) |
| unknown | 0 (0.0) | 0 (0.0) | 1 (1.6) | 1 (1.8) | 1 (2.6) | 0 (0.0) | 1 (5.0) | 0 (0.0) | 0 (0.0) | 0 (0.00 | 0 (0.00 | 0 (0.0) | 0 (0.0) | 2 (5.6) | 0 (0.0) |
| <20 | 8 (5.7) | 3 (3.1) | 2 (3.3) | 3 (5.3) | 1 (2.6) | 0 (0.0) | 0 (0.0) | 0 (0.0) | 0 (0.0) | 0 (0.0) | 2 (2.3) | 0 (0.0) | 0 (0.0) | 0 (0.0) | 1 (3.7) |
| 20-39 | 57 (41.4) | 18(18.7) | 17 (27.9) | 21 (36.8) | 9 (23.1) | 0 (0.0) | 13 (65.0) | 2 (15.4) | 4 (25.0) | 1 (10.0) | 2 (2.3) | 4 (17.4) | 3 (8.6) | 10 (27.8) | 7 (25.9) |
| 40-59 | 58 (41.4) | 36 (37.5) | 36 (59.0) | 18 (31.6) | 18 (46.2) | 10 (27.0) | 3 (15.0) | 5 (38.5) | 8 (50.0) | 5 (50.0) | 25 (28.7) | 4 (17.4) | 5 (14.3) | 17 (47.2) | 12 (44.4) |
| 60-79 | 9 (6.38) | 37 (38.5) | 4 (6.5) | 5 (8.8) | 9 (23.1) | 23 (62.1) | 3 (15.0) | 5 (38.5) | 4 (25.0) | 3 (30.0) | 38 (43.7) | 13 (56.5) | 20 (57.1) | 9(25.0) | 5 (18.5) |
| ≥ 80 years | 0 (0.0) | 2 (2.1) | 0 (0.0) | 2 (3.5) | 1 (2.6) | 2 (5.4) | 0 (0.0) | 1 (7.7) | 0 (0.0) | 0 (0.0) | 17 (19.5) | 2 (8.7) | 4 (11.4) | 0 (0.0) | 0 (0.0) |
| Unknown | 9 (6.4) | 0 (0.0) | 2 (3.3) | 8 (14.0) | 1 (2.6) | 2 (5.4) | 1 (5.00) | 0 (0.0) | 0 (0.0) | 1 (10.0) | 3 (3.5) | 0 (0.0) | 3 (8.6) | 0 (0.0) | 2 (7.4) |
| AP treatment at the time of report (all formulations), n (%) | | | | | | | | | | | | | | | |
| N | 141 | 94 | 61 | 57 | 39 | 33 | 19 | 13 | 14 | 10 | 79 | 19 | 33 | 33 | 20 |
| AP monotherapy | 57(40.4) | 41(43.6) | 26(42.6) | 20(35.1) | 15(38.5) | 15(45.5) | 9(47.4) | 10(76.9) | 8(57.1) | 6(60.0) | 66(83.5) | 15(78.9) | 21(63.6) | 17(51.5) | 9(45.0) |
| AP polytherapy | 84(59.6) | 53(56.4) | 35(57.4) | 37(64.9) | 24(61.5) | 18(54.5) | 10(52.6) | 3(23.1) | 6(42.9) | 4(40.0) | 13(16.5) | 4(21.1) | 12(36.4) | 16(48.5) | 11(55.0) |
|  |  |  |  |  |  |  |  |  |  |  |  |  |  |  |  |
| Any FGA | 43(30.5) | 31(33.0) | 13(21.3) | 24(42.1) | 10(25.6) | 5(15.2) | 7(36.8) | 0(0.0) | 2(14.3) | 4(40.0) | 50(63.3) | 11(57.9) | 20(60.6) | 21(63.6) | 8(40.0) |
| Any SGA | 139(98.6) | 87(92.6) | 60(98.4) | 55(96.5) | 39(100.0) | 32(97.0) | 18(94.7) | 13(100.0) | 14(100.0) | 8(80.0) | 36(45.6) | 11(57.9) | 16(48.5) | 18(54.5) | 16(80.0) |
| Group1 | 63(45.3) | 32(36.8) | 31(51.7) | 30(54.5) | 17(43.6) | 22(68.8) | 10(55.6) | 9(69.2) | 6(42.9) | 3(37.5) | 13(36.1) | 4(36.4) | 4(25.0) | 5(27.8) | 5(31.3) |
| Group2 | 76(54.7) | 58(66.7) | 32(53.3) | 35(63.6) | 30(76.9) | 8(25.0) | 5(27.8) | 1(7.7) | 4(28.6) | 0(0.0) | 13(36.1) | 4(36.4) | 3(18.8) | 1(5.6) | 3(18.8) |
| Group3 | 45(32,4) | 11(12.6) | 9(15.0) | 19(34.5) | 6(15.4) | 12(37.5) | 7(38.9) | 2(15.4) | 3(21.4) | 3(37.5) | 8(22.2) | 1(9.1) | 5(31.3) | 7(38.9) | 6(37.5) |
| Group4 | 78(56.1) | 27(31.0) | 13(21.7) | 27(49.1) | 12(30.8) | 9(28.1) | 6(33.3) | 4(30.8) | 7(50.0) | 4(50.0) | 10(27.8) | 4(36.4) | 7(43.8) | 9(50.0) | 9(56.3) |
| AP treatment at the time of report (oral and injectable formulation only) | | | | | | | | | | | | | | | |
| N | 130 | 90 | 58 | 55 | 39 | 31 | 19 | 13 | 10 | 10 | 54 | 16 | 29 | 30 | 16 |
| AP monotherapy | 51(39.2) | 39(43.3) | 24(41.4) | 19(34.5) | 15(38.5) | 13(41.9) | 9(47.4) | 10(76.9) | 6(60.0) | 6(60.0) | 44(81.5) | 12(75.0) | 19(65.5) | 16(53.3) | 7(43.8) |
| AP polytherapy | 79(60.8) | 51(56.7) | 34(58.6) | 36(65.5) | 24(61.5) | 18(58.1) | 10(52.6) | 3(23.1) | 4(40.0) | 4(40.0) | 10(18.5) | 4(25.0) | 10(34.5) | 14(46.7) | 9(56.3) |
|  |  |  |  |  |  |  |  |  |  |  |  |  |  |  |  |
| Any FGA | 36(27.7) | 27(30.0) | 12(20.7) | 22(40.0) | 9(23.1) | 5(16.1) | 6(31.6) | 0(0.0) | 2(20.0) | 4(40.0) | 34(63.0) | 7(43.8) | 17(58.6) | 17(56.7) | 5(31.3) |
| Any SGA | 127(97.7) | 83(92.2) | 57(98.3) | 53(96.4) | 39(100.0) | 30(96.8) | 18(94.7) | 13(100.0) | 10(100.0) | 8(80.0) | 26(48.1) | 11(68.8) | 14(48.3) | 18(60.0) | 13(81.3) |
| Group1 | 61(48.0) | 30(36.1) | 30(52.6) | 27(50.9) | 17(43.6) | 21(70.0) | 10(55.6) | 9(69.2) | 5(50.0) | 3(37.5) | 9(34.6) | 4(36.4) | 4(28.6) | 5(27.8) | 4(30.8) |
| Group2 | 70(55.1) | 51(61.4) | 26(45.6) | 33(62.3) | 29(74.4) | 8(26.7) | 4(22.2) | 1(7.7) | 1(10.0) | 0(0.0) | 8(30.8) | 3(27.3) | 2(14.3) | 0(0.0) | 2(15.4) |
| Group3 | 42(33.1) | 10(12.0) | 8(14.0) | 18(34.0) | 6(15.4) | 11(36.7) | 6(33.3) | 2(15.4) | 1(10.0) | 3(37.5) | 8(30.8) | 1(9.1) | 4(28.6) | 6(33.3) | 5(38.5) |
| Group4 | 71(55.9) | 26(31.3) | 11(19.3) | 25(47.2) | 12(30.8) | 7(23.3) | 6(33.3) | 4(30.8) | 5(50.0) | 4(50.0) | 8(30.8) | 4(36.4) | 6(42.9) | 9(50.0) | 6(46.2) |

Sup.Table.2 The five most reported AEs among the EPS

Abbreviation: AP, antipsychotic; D2R, dopamine receptor 2 partial antagonist; SCH, schizophrenia; Group1: antagonist of dopamine and serotonin, Group2: antagonist of dopamine, serotonin, and norepinephrine, Group3: multimodal agent of dopamine and serotonin and norepinephrine, Group4: partial agonist of dopamine and serotonin
